# Spin-Resolved Dielectric Bath Embedding Theory for Metallic Surfaces

**DOI:** 10.1021/acs.jctc.6c01355

**Published:** 2026-08-24

**Authors:** Kwanpyung Lee, Connor Fawcett, Qing Zhao

**Affiliations:** Department of Chemical Engineering, 1848 Northeastern University, Boston, Massachusetts 02115, United States

## Abstract

We introduce spin-resolved dielectric bath embedding theory (SR-DBET), which incorporates a physics-informed embedding potential to describe attractive and repulsive interactions, a charge dielectric bath to capture charge polarization, and a spin dielectric bath to account for spin density fluctuations. We demonstrate the accuracy of SR-DBET through adsorption energy calculations on Cu surfaces. This embedding scheme is essential for extending embedded correlated wave function theory to spin-polarized quantum systems.

## Graphical abstract

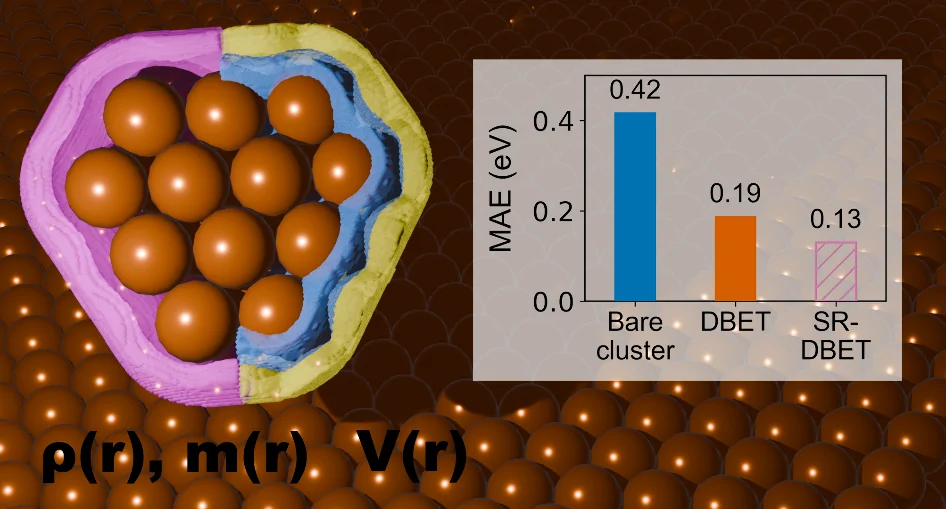

Quantum embedding approaches enable accurate descriptions of local electronic structure in large systems.^1–5^ In these methods, the full system is usually partitioned into a local region of interest, often denoted as a cluster, and its surrounding environment. The cluster is treated at high-level correlated wave function (CW) theory, while its interaction with the environment is described using an embedding scheme tailored to the material. For example, orbital-partitioning-based density matrix embedding theory^6–9^ and its variants, such as bootstrap embedding,^10–12^ provide a rigorous treatment of the entanglement through bath orbitals and have been applied to systems with finite band gaps and graphene-like systems.^9,10,13^ Alternatively, density-partitioning-based embedding schemes for metallic systems, such as the density functional embedding theory (DFET),^2,14,15^ employ an embedding potential to reproduce the electron densities of the subsystems. More recently, we developed dielectric bath embedding theory (DBET),^16^ which combines a physics-informed embedding potential to approximate attractive and repulsive interactions with a charge dielectric bath to capture adsorbate-induced charge polarization (Figure 1).

**Figure 1:**
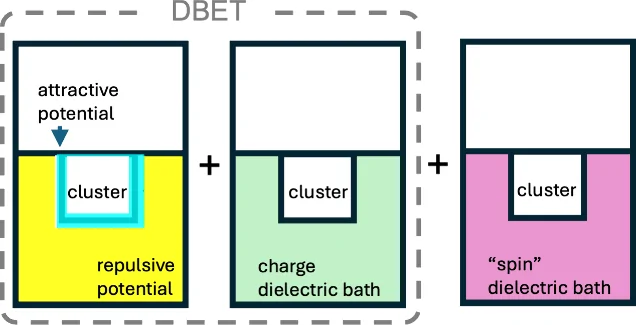
Schematics of DBET and spin-resolved DBET (SR-DBET). In DBET, a cluster is immersed in an external local potential (left; cyan for attractive potential and yellow for repulsive potential) and a charge dielectric bath (middle; green). In SR-DBET, an additional spin dielectric bath (right; magenta) is introduced. External local potential, charge dielectric bath, and spin dielectric bath are extended in the region of the environment.

The motivation for developing DBET stems from the observation that reproducing the electron density alone may not be sufficient to reproduce the physical properties of the extended surface. In particular, the correlation energy derived from CW methods depends on the virtual orbital space, and it remains unclear whether the virtual orbitals interact appropriately with the density-based embedding potentials within the framework of embedded correlated wave function (ECW) theory. Additionally, the embedding potential is optimized to reproduce electron density at the DFT level, whereas subsequent high-level CW calculations might yield a different electron density. These considerations motivate the development of an embedding scheme based on physical insights rather than density matching alone. Specifically, DBET incorporates a charge dielectric bath that responds to changes in the electron density induced by local environment, such as adsorbates or solvents, motivated by the infinite dielectric constant of an ideal conductor.^16^ In addition, the embedding potential in DBET consists of an attractive potential in the boundary region and a repulsive potential in the surrounding region. Compared with conventional density-based embedding methods, DBET explicitly accounts for environmental dielectric screening of local charge fluctuations while substantially improving the computational efficiency of embedding potential optimization. We recently showed that DBET outperforms DFET in predicting Fermi level, charge states, and adsorption energies of a variety of adsorbates on Cu surfaces.^16^ The improvement in predicting binding strengths is most pronounced for polar adsorbates, where the induced electron density fluctuations of the cluster can be appropriately handled with the charge dielectric bath in DBET. In contrast, nonpolar adsorbates usually produce weaker charge density fluctuations but can induce significant spin density fluctuations, which cannot be properly captured by DBET.

To illustrate the limitations of DBET, as well as DFET, arising from their inability to capture spin density fluctuations induced by adsorbates, we compared the spin density changes of a Cu_18_ cluster upon adsorption of three representative adsorbates within the DBET framework: CO at the face-centered cubic (fcc) site, where DBET performs poorly; CO at the top site, where DBET exhibits moderate accuracy, and NH_3_ at the top site, where DBET performs well (Figure 2). All calculations were carried out at the DFT level with the Perdew–Burke–Ernzerhof (PBE) exchange correlation (XC) functional^17^ using the Vienna Ab initio Simulation Package (VASP) version 6.3.2.^18–20^ (Supporting Information or SI Supplementary Method). A clear correlation is observed between the spin density fluctuations due to the adsorbates and the corresponding errors in predicted adsorption energies relative to the periodic slab calculations, motivating the development of a spin-resolved embedding scheme capable of capture spin density fluctuations. We note that although Cu is a nonmagnetic metal, magnetization or spin polarization can emerge in a Cu cluster due to the breaking metallic bonds at the boundary between the cluster and its environment. A physically realistic embedding scheme should therefore stabilize the boundary spin density to mimic the extended metallic environment while remaining dynamically responsive to spin density fluctuations induced by adsorbates.

**Figure 2:**
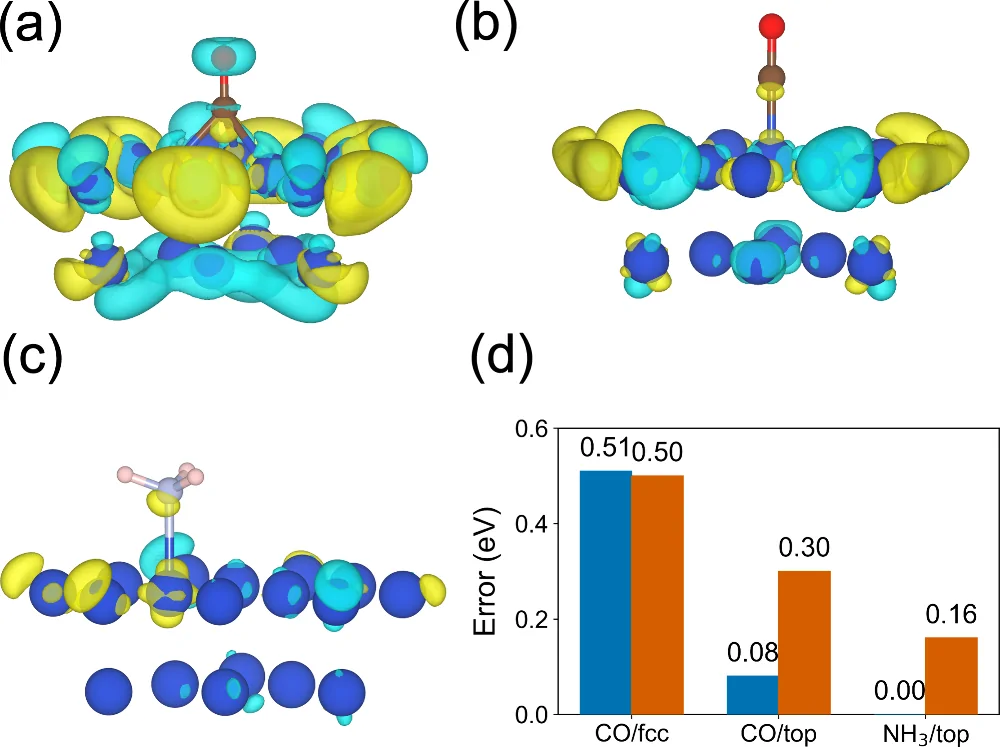
Changes in spin density of a Cu_18_ cluster upon adsorption of (a) a CO adsorbed at the fcc site, (b) a CO adsorbed at the top site, and (c) a NH_3_ adsorbed at the top site within the DBET framework. (d) Errors in adsorption energies predicted by an embedded Cu_18_ cluster using DBET (blue bars) and DFET (orange bars), referenced against the corresponding periodic slab calculations. Yellow and cyan isosurfaces represent spin up and spin down density differences, with an isosurface level of 10^–9^ e/Å^3^. Atoms are colored as follows: blue for Cu, brown for C, red for O, blue for N, and light pink for H.

Before introducing our methodology, we first outline the key requirements for a spin-polarized embedding scheme applicable to metallic systems: (i) proper stabilization of the spin density in the boundary region; (ii) a short-range embedding interaction that avoids spurious interactions with the adsorbates; and (iii) a systematic and transferable construction of the embedding potential that is generalizable to different adsorbates and metal surfaces. Standard DFET does not satisfy the first two requirements because the reference electron density used to optimize the embedding potential is spin-independent, and the resulting embedding potential can extend into the active site and adsorbate regions.^16^ In contrast, DBET substantially improves the second requirement that minimizes interactions between embedding potential and the adsorbate, but it does not consider spin density effect. Here, we introduce spin-resolved DBET (SR-DBET), which extends DBET by incorporating a spin density-dependent embedding potential derived from a spin dielectric bath to address spin density fluctuations.

The spin density-dependent potential in SR-DBET is constructed by modifying the polarizable continuum model (PCM),^21^ in which the amplified spin density (scaled by a scaling factor, C), rather than the charge density, serves as the source term, and the Coulomb kernel is replaced by the Yukawa kernel to ensure a short-range interaction and to avoid spurious long-range interactions with the adsorbate,

$$K\left(r,r^{\prime }\right)=\frac{e^{-\kappa \left|r-r\prime \right|}}{\left|r-r^{\prime }\right|}$$

in which κ is the screening parameter. The spin density is first multiplied by a scaling factor and used as the source term to generate the surface potential *v*. The corresponding surface charges, *q*, are then determined by solving the modified PCM matrix equation,

Kq=Rv

where *K* is the Yukawa surface–surface interaction matrix and *R* is the dielectric response matrix. The resulting spin bath potential, ϕ_*spin bath*_, is evaluated as the Yukawa potential generated by the induced surface effective charge. This potential is subsequently added to the α Fock matrix and subtracted from the β Fock matrix (assuming the α channel is the majority spin channel), thereby stabilizing the majority spin orbitals:

$$\begin{array}{l} \phi^{\alpha }\left(r\right)=\phi_{KS}+\phi_{DBET}+\phi_{spin\;bath}\\ \phi^{\beta }\left(r\right)=\phi_{KS}+\phi_{DBET}-\phi_{spin\;bath} \end{array}$$

Additional details of the PCM can be found elsewhere.^22,23^ Here, the PCM cavity is modified following the original DBET formulation such that it is constructed exclusively within the environment region. The SR-DBET parameters were optimized using hydrogen adsorption at the hollow site on Cu, yielding κ = 3.5, C = 21, and a spin dielectric constant of 100. The parameters for the external embedding potential and the charge dielectric bath were retained from the original DBET, except for the coefficient of the attractive potential, which was reduced from 10.5 to 7 since the spin bath also contributes to stabilizing the boundary region. Here, we note that the charge dielectric bath was implemented using the standard PCM with the Coulomb kernel and the same modified cavity. The differences between the two baths therefore arise from both the source term (charge vs spin) and the kernel (Coulomb vs Yukawa). Applications of SR-DBET to different metallic systems may require reoptimization and validation of the dielectric bath parameters. A detailed description of the SR-DBET formalism is provided in the SI Supplementary Method. The implementation of SR-DBET is available in a modified PySCF module,^24,25^ which is available through Github.^26^

We next demonstrate the improvement of SR-DBET in predicting adsorption energies for the same set of 12 adsorbates on Cu(111) surfaces considered in our original DBET work, arising from its explicit treatment of spin density. The data set includes H, C, N, O, Cl, Na, CH, CO, NH, and OH adsorbed at fcc sites and H_2_O, NH_3_, and CO adsorbed at top sites. Multiple adsorbate–surface distances were considered for each system, resulting in a total of 80 adsorption energy data points, consistent with the original DBET study. Embedded cluster calculations with SR-DBET were performed using the PBE XC functional^17^ and Gaussian-type orbital (GTO) basis sets, cc-pVDZ-PP^27^ for Cu (with the corresponding effective core potential replacing the ten core electrons) and aug-cc-pVDZ^28,29^ for the adsorbates, using a Cu_18_ cluster implemented in our modified PySCF.^24,25^ Periodic slab calculations, bare cluster calculations, and embedded cluster calculations with DFET and the original DBET were taken from our previous work, in which the PBE XC functional and plane-wave (PW) basis sets were employed. To account for the basis set difference between PW and GTO calculations, we corrected the SR-DBET errors by adding the corresponding energy differences obtained from bare cluster calculations with the two basis sets. Additional computational details are provided in the SI Supplementary Method.

As expected, SR-DBET predicts a nonzero magnetization of 4 μ_*B*_ as the ground state of the embedded Cu_18_ cluster (Figure 3). The spin density is localized primarily at the cluster-environment boundary region, indicating that the magnetization originates from the breaking of metallic bonds at the cluster boundary. The spin bath potential generated by SR-DBET stabilizes this boundary spin density while confining it to the boundary region. For adsorption energy predictions, SR-DBET performs remarkably better than both DFET and the original DBET, reducing the mean absolute error (MAE) from 0.34 eV for DFET and 0.19 eV for DBET to 0.13 eV, using periodic slab calculations as the reference (Figure 4). The improvement is particularly significant for nonpolar adsorbates (H, C, CH, and CO), for which the MAE decreases from 0.30 eV with DBET to 0.14 eV with SR-DBET. As a result, SR-DBET achieves a more balanced level of accuracy for both polar and nonpolar adsorbates, demonstrating its robustness as an embedding scheme for metallic surfaces.

**Figure 3:**
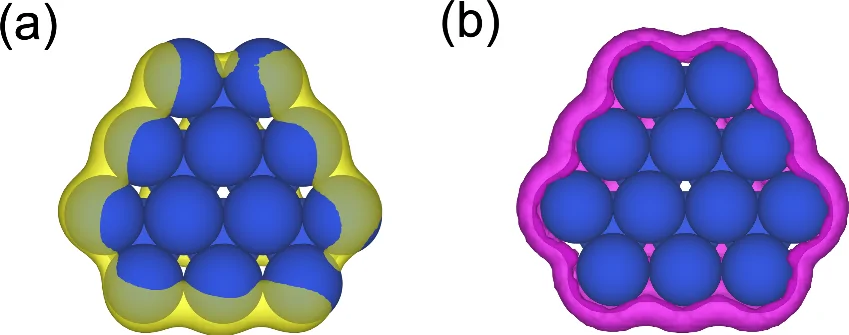
(a) Spin density distribution of the embedded Cu_18_ cluster predicted by SR-DBET. (b) Spin potential generated by the spin dielectric bath in SR-DBET.

**Figure 4:**
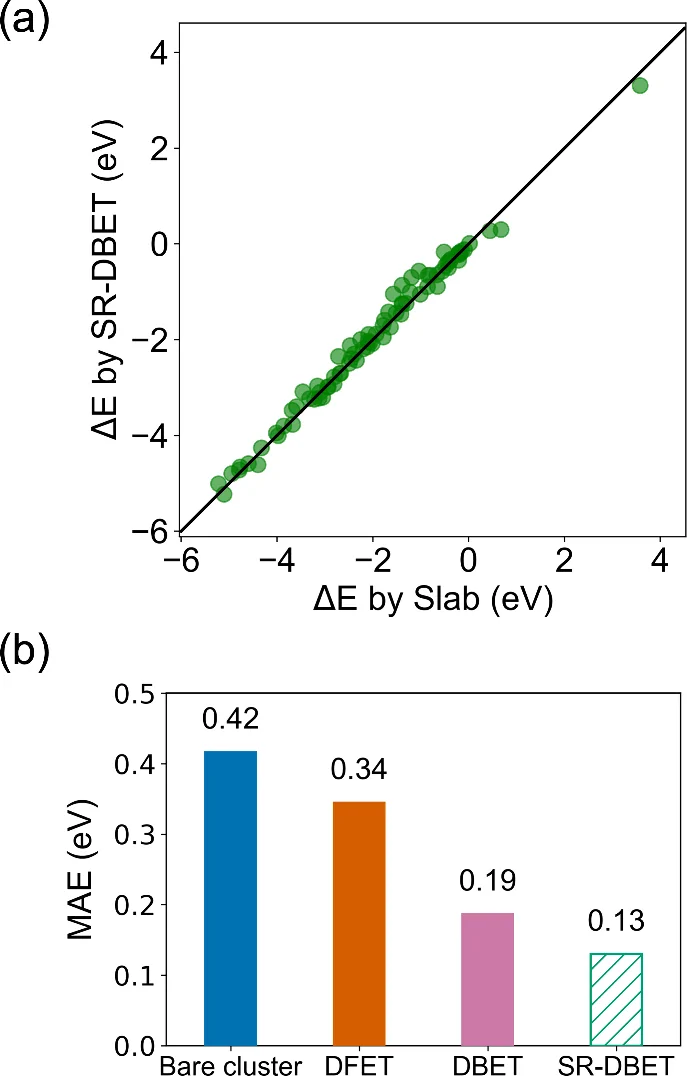
(a) Parity plot of adsorption energies for 12 adsorbates on Cu(111) at multiple adsorbate–surface distances, predicted by embedded cluster calculations with SR-DBET, referenced against periodic slab calculations. For each adsorbate, five adsorbate–surface distances were considered, except for H, Cl, and Na, for which ten distances were evaluated. Two adsorption sites were considered for CO. In total, 80 data points were included here. (b) A bar chart showing the mean absolute errors (MAE) in eV for the bare cluster calculations, embedded cluster calculations with DFET, DBET, and SR-DBET.

In conclusion, our work demonstrates that an embedding scheme must dynamically respond to changes in both the charge density and spin density of the cluster to reproduce the electronic behavior of the environment of the corresponding extended surface. Building upon DBET, which employs a charge dielectric bath to respond to changes in charge density, we introduce SR-DBET by incorporating a spin dielectric bath to capture spin density fluctuations induced by the local environment. We show that SR-DBET consistently outperforms the original DBET in predicting adsorption energies of various adsorbates on Cu surfaces, with particularly significant improvements for nonpolar adsorbates. Here, we used the Cu surface as the benchmark because it is the most extensively studied metallic surface within the ECW framework. We are currently extending SR-DBET to other catalytically relevant metallic systems, including Pt and magnetic Fe. The optimal charge and spin dielectric bath parameters may require further optimization and validation for different materials. We believe that SR-DBET represents an accurate and computationally efficient embedding scheme for coupling with multireference wave function theories to enable reliable predictions of properties that are challenging for conventional DFT in heterogeneous catalysis, including reaction barriers and complex electronic structure properties. By improving the description of the embedding potential, SR-DBET is designed to minimize embedding errors, allowing the accuracy of high-level electronic structure methods to be more faithfully preserved.

## Supplementary Material



## Data Availability

The data underlying this study are available in the published article and the Supporting Information. The code used to generate and analyze the results is available at Github: https://github.com/qingzhao-neu/Dielectric-bath-embedding-theory-DBET-.

## Notes

Additional details on the formalism and implementation of SR-DBET, as well as computational methods (PDF)
