# Supplementary material for: Spin-Resolved Dielectric Bath Embedding Theory for Metallic Surfaces
Source: J Chem Theory Comput. 2026 Aug 24;22(17):8725–9. doi: 10.1021/acs.jctc.6c01355 (PMC13564350; doi:10.1021/acs.jctc.6c01355)
Supplement: Supplementary file 1 [file ct6c01355_si_001.pdf]

Supporting Information for

Spin-Resolved Dielectric Bath Embedding Theory for Metallic Surfaces

Kwanpyung Lee, Connor Fawcett, and Qing Zhao*

Department of Chemical Engineering, Northeastern University, Boston, MA 02115, United States.

***Corresponding author:** Qing Zhao (Email: q.zhao@northeastern.edu)

Contents

Supplementary Method. SR-DBET implementation and computational methods Page S2

Figure S1. Schematic illustration of the cavity surface construction. Page S7

Figure S2. Example of the cavity surface within the cluster. Page S8

Supplementary Method. SR-DBET implementation and computational methods.

Modification of the cavity construction. We modified the cavity construction so that the cavity represents the interaction between the cluster and its surroundings. In this representation, the cavity surface above the metallic cluster is not supposed to interact with the cluster. Therefore, we define the surface boundary as the position of the top layer plus half of the interlayer distance. For example, the boundary is set to 9.28 Å (8.24+0.5×2.07), where the center of the top layer is at 8.24 Å and the interlayer distance is 2.07 Å (Figure S1). The cavity surface grid points above this boundary have zero switch_fun (switching function) and thus contribute no induced surface charge to the reaction-field potential.

In addition, the default atomic radius for cavity construction undesirably places cavity surface grid points inside the metallic cluster (Figure S2). Therefore, the radius for Cu was set to 1.82 Å to ensure sufficient overlap between neighboring atomic spheres. Furthermore, the switching width parameter R_sw_J was redefined as "R_sw_J = R_J · (0.250 / N_J)**0.5", where N_J is the number of Lebedev grid points on sphere J.

Implementation of spin dielectric bath. The spin dielectric bath is implemented as a custom solvation module built on top of PySCF's standard PCM framework. The central equation has the same form as C-PCM:

$$\boldsymbol{Kq} = \boldsymbol{Rv}$$

where **q** is the induced surface charges, **v** is the surface potential, **K** is the surface-surface interaction matrix, and **R** is the dielectric response matrix. Below we describe each component in parallel with its standard PCM counterpart, highlighting where and why this work differs.

Source of the Surface Potential

	Standard PCM	This work
source	electron density	spin density

In standard PCM, the surface potential **v** is the electrostatic potential of the electron density at each surface point, evaluated via analytic integral methods. In this work, the source is instead the spin density evaluated numerically on the DFT quadrature grid:

$$v_i = \sum_g T_{gi} C w_g m_g$$

where T_{gi} is the grid-surface Yukawa kernel (see below), C is an empirical prefactor, and w_g and m_g are the quadrature weights and the spin density, respectively.

Surface-Surface Kernel

	Standard PCM	This work
Kernel	Coulomb $\mathrm{erf}(\xi_{ij} r_{ij})/r_{ij}$	Yukawa $\mathrm{erf}(\xi_{ij} r_{ij})/r_{ij} \cdot e^{-\kappa r_{ij}}$
off-diagonal	$\mathrm{K}_{ij} = \mathrm{erf}(\xi_{ij} r_{ij})/r_{ij}$	$\mathrm{K}_{ij} = \mathrm{erf}(\xi_{ij} r_{ij})/r_{ij} \cdot e^{-\kappa r_{ij}}$
diagonal	$\mathrm{K}_{ii} = \mathrm{S}_{ii} = \sqrt{2/\pi}\xi_i$	$\mathrm{K}_{ii} \approx S_{ii} - \kappa$

In both cases, $\xi_{ij} = \xi_i \xi_j / \sqrt{\xi_i^2 + \xi_j^2}$ is the combined Gaussian smearing exponent inherited from the surface construction, which regularizes the short-range divergence of the Coulomb kernel. Physically, the surface charges are point-like (i.e., delta functions), and the Gaussian smearing is a numerical device that becomes exact in the limit $\xi_i \to \infty$.

For the off-diagonal elements, the Yukawa screening factor $e^{-\kappa r_{ij}}$ is evaluated at the center-to-center distance, r_{ij}. In the limit where the two surface charge distributions are effectively point-like relative to their separation, this is exact; the Gaussian regularization corrects for finite Gaussian width in the same manner as standard PCM, introducing no approximation beyond what is already present in the $\kappa = 0$ case.

The diagonal requires a separate treatment. In standard PCM, the self-potential of the smeared Gaussian has an analytic form. For the Yukawa case, the exact self-interaction:

$$K_{ii}^{exact} = \int d\boldsymbol{r}\, \rho_i(\boldsymbol{r}) \frac{e^{-\kappa r}}{\boldsymbol{r}} \mathrm{erf}(\xi_i \boldsymbol{r})$$

has no closed form at $\kappa \neq 0$. A first-order Taylor expansion in κ gives $\mathrm{K}_{ii} \approx S_{ii} - \kappa$, which captures the leading effect of Yukawa screening on the self-interaction.

Grid–Surface Kernel Matrix

	Standard PCM	This work
Fock matrix	Analytic 3-center integrals	Numerical via precomputed **T**

Kernel	Coulomb $\text{erf}(\xi_{ij}r_{ij})/r_{ij}$	Yukawa $\text{erf}(\xi_{ij}r_{ij})/r_{ij} \cdot e^{-\kappa r_{ij}}$

Standard PCM evaluates the Fock matrix contribution from surface charges using analytic 3-center electron repulsion integrals. In this work, the screened Yukawa potential has no analytic integral representation in a Gaussian basis, so the contribution is instead evaluated numerically via a precomputed grid-surface kernel matrix:

$$T_{gi} = \text{erf}(\xi_i r_{gi})/r_{gi} \cdot e^{-\kappa r_{gi}}$$

This matrix of dimension $N_{grid} \times N_{surf}$ is computed once and used for surface potential calculation and Fock matrix construction.

Self-Consistency Scheme

	Standard PCM	This work
PCM update frequency	Every SCF micro-iteration	Once per macro-iteration (outer loop)

In standard PCM, the surface charges are updated at every SCF micro-iteration. In this work, a macro-iteration strategy is employed instead: the inner SCF converges with a frozen Fock contribution after which the surface charges are recomputed from the converged density matrix. This outer loop repeats until $max|\Delta V_{ij}| < tol$ (default: 10^{-6} Ha), typically requiring 3-8 outer iterations. Furthermore, the macro-iteration scheme is naturally compatible with higher-level correlated methods: the converged spin dielectric bath potential from a mean-field calculation can be passed directly as a frozen one-body contribution to the correlated calculation, without requiring any further update of the surface charges.

Computational methods. The periodic calculations and the corresponding bare cluster calculations were taken from our previous study.[1] We used the same DBET parameters to analyze the changes in spin distribution shown in Figure 2 of the main text. For completeness, these calculations were performed using VASP.6.3.2.[2-4] We used the Perdew-Burke-Ernzerhof (PBE) XC functional, and the core electrons are described using the projector-augmented wave (PAW) method.[5] A kinetic energy cutoff of 500 eV was used for the plane wave basis set. Also, Γ-point-

only Monkhorst-Pack grids were used for all cluster calculations, while a 3×3×1 k-point mesh was used for the four-layered 6×6 slab to sample the Brillouin zone.

On the other hand, as the spin density is treated as charge in SR-DBET, the electronic energy of a system under periodic boundary conditions with nonzero net charge becomes size-dependent. To avoid this artifact, all SR-DBET calculations were performed with computational chemistry software without periodic boundary conditions using PySCF[6,7] with additional modules to implement the features of SR-DBET, such as reading embedding potential, modification of the cavity, and the implementation of the spin dielectric bath, which are available on Github. The PBE functional[8] was employed with a cc-pVDZ-PP basis set[9] for Cu and an aug-cc-pVDZ basis set[10-12] for the adsorbates. The error was evaluated using our previous calculations from the planewave periodic DFT code VASP[2-4], which was employed for the periodic slab calculations:

$$Error = \Delta E_{SR-DBET}^{PySCF} - \Delta E_{Slab}^{VASP} + (\Delta E_{Bare\ cluster}^{VASP} - \Delta E_{Bare\ cluster}^{PySCF})$$

Note that we confirmed that bare cluster calculations using PySCF and VASP are in good agreement, with a mean absolute deviation of less than 0.02 eV.

SR-DBET optimization timing. The computational advantage of SR-DBET arises because, once the single-point calculations of the cluster, environment, and full slab are performed to generate the external potentials, only the cluster is considered during the optimization of the dielectric bath parameters. In contrast, DFET requires an iterative L-BFGS optimization of the embedding potential, with repeated cluster and environment calculations until convergence. For the Cu system studied here, a single SR-DBET cluster calculation requires approximately 20-30 minutes on a Cascade Lake node. By comparison, the DFET embedding potential optimization requires approximately six days and four hours of wall time on the same node, with an L-BFGS convergence threshold of $3 \cdot 10^{-4}$ Å^{-3}.

The most computational demanding step in SR-DBET is the optimization of the dielectric bath parameters. This process first requires obtaining the charge densities of the cluster, environment, and reference full slab to construct the attractive and repulsive potentials. This one-time step took approximately two hours for the Cu system studied here on the same Cascade Lake node. Parameter optimization then requires DFT calculations for 11 structures corresponding to *H adsorption on Cu(111), including the clean cluster and H adsorbates at different adsorption sites. Each calculation requires 20-30 minutes, resulting in a total wall time of approximately four

hours and 30 minutes per optimization iteration. In practice, parameter optimization typically requires fewer than 20 iterations and can therefore be completed within approximately four days when performed serially, which is still shorter than a single DFET embedding potential optimization. Each iteration is independent and can therefore be evaluated simultaneously. In practice, multiple candidate configurations were evaluated in parallel during the optimization, substantially reducing the overall wall time. Our ongoing work focuses on developing a more systematic and automated SR-DBET parameter optimization protocol and establishing optimized parameters for most of the representative catalytic metal surfaces.

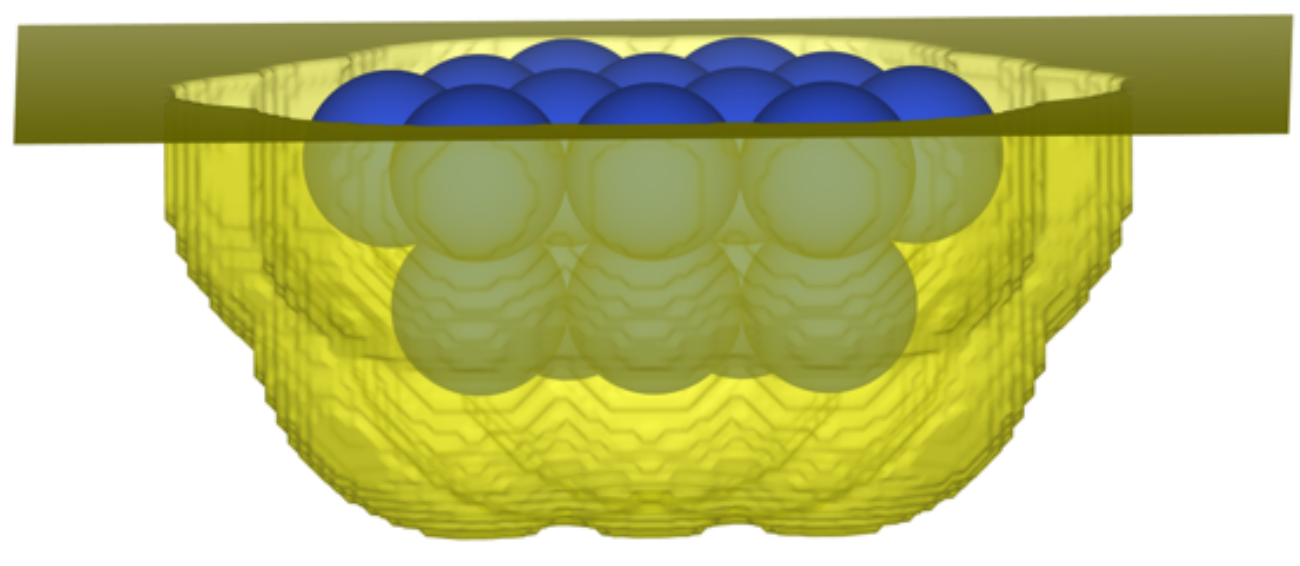

Figure S1. A schematic illustration of the cavity surface construction.

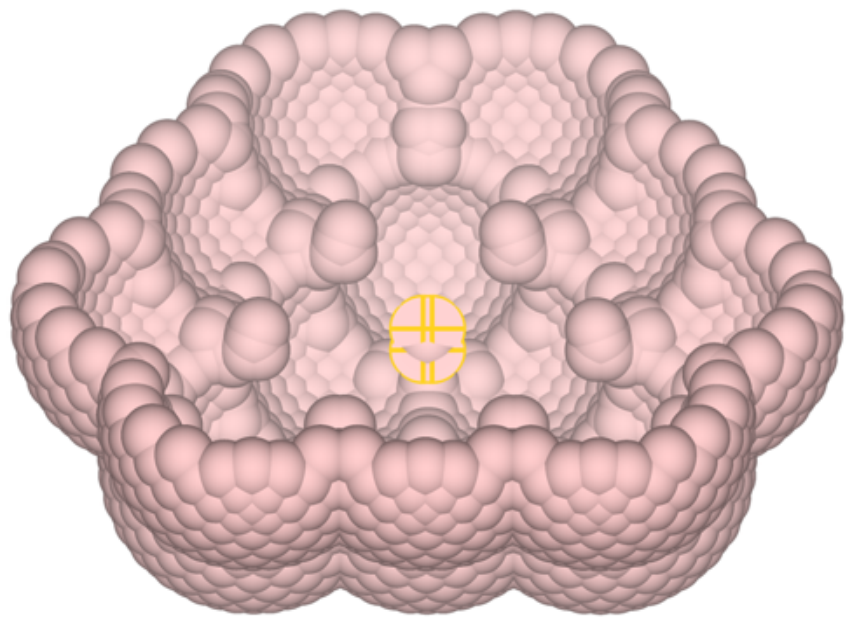

Figure S2. Example of the cavity surface grid points (yellow crosses and circles) within the cluster.

References

(1) Lee, K.; Fawcett, C.; Zhao, Q. Introducing a dielectric bath embedding theory for embedded electronic structure calculations in heterogeneous catalysis. *The Journal of Chemical Physics* **2026**, *164*, 114104.
(2) Kresse, G.; Furthmüller, J. Efficiency of ab-initio total energy calculations for metals and semiconductors using a plane-wave basis set. *Computational Materials Science* **1996**, *6* (1), 15-50.
(3) Kresse, G.; Furthmüller, J. Efficient iterative schemes for ab initio total-energy calculations using a plane-wave basis set. *Physical Review B* **1996**, *54* (16), 11169.
(4) Kresse, G.; Hafner, J. Ab initio molecular dynamics for liquid metals. *Physical Review B* **1993**, *47* (1), 558.
(5) Kresse, G.; Joubert, D. From ultrasoft pseudopotentials to the projector augmented-wave method. *Physical Review B* **1999**, *59* (3), 1758.
(6) Sun, Q.; Berkelbach, T. C.; Blunt, N. S.; Booth, G. H.; Guo, S.; Li, Z.; Liu, J.; McClain, J. D.; Sayfutyarova, E. R.; Sharma, S. PySCF: the Python-based simulations of chemistry framework. *Wiley Interdisciplinary Reviews: Computational Molecular Science* **2018**, *8* (1), e1340.
(7) Sun, Q.; Zhang, X.; Banerjee, S.; Bao, P.; Barbry, M.; Blunt, N. S.; Bogdanov, N. A.; Booth, G. H.; Chen, J.; Cui, Z.-H. Recent developments in the PySCF program package. *The Journal of Chemical Physics* **2020**, *153* (2), 024109.
(8) Perdew, J. P.; Burke, K.; Ernzerhof, M. Generalized gradient approximation made simple. *Physical Review Letters* **1996**, *77* (18), 3865.
(9) Peterson, K. A.; Figgen, D.; Dolg, M.; Stoll, H. Energy-consistent relativistic pseudopotentials and correlation consistent basis sets for the 4d elements Y–Pd. *The Journal of Chemical Physics* **2007**, *126* (12).
(10) Dunning Jr, T. H. Gaussian basis sets for use in correlated molecular calculations. I. The atoms boron through neon and hydrogen. *The Journal of Chemical Physics* **1989**, *90* (2), 1007-1023.
(11) Kendall, R. A.; Dunning Jr, T. H.; Harrison, R. J. Electron affinities of the first-row atoms revisited. Systematic basis sets and wave functions. *The Journal of Chemical Physics* **1992**, *96* (9), 6796-6806.
(12) Woon, D. E.; Dunning Jr, T. H. Gaussian basis sets for use in correlated molecular calculations. III. The atoms aluminum through argon. *The Journal of Chemical Physics* **1993**, *98* (2), 1358-1371.
